# Gender differences in suicidal expressions and their determinants among young people in Cambodia, a post-conflict country*

**DOI:** 10.1186/1471-244X-11-47

**Published:** 2011-03-21

**Authors:** Bhoomikumar Jegannathan, Gunnar Kullgren

**Affiliations:** 1 Center for Child and Adolescent Mental Health, Chey Chumneas Hospital, Cambodia; 2 Division of Psychiatry, Department of Clinical Sciences, University of Umeå, Sweden

## Abstract

**Background:**

Suicide among young people is a global public health problem, but adequate information on determinants of suicidal expression is lacking in middle and low income countries. Young people in transitional economies are vulnerable to psychosocial stressors and suicidal expressions. This study explores the suicidal expressions and their determinants among high school students in Cambodia, with specific focus on gender differences.

**Methods:**

A sample of 320 young people, consisting of 153 boys and 167 girls between 15-18 years of age, was randomly selected from two high schools in Cambodia. Their self-reported suicidal expressions, mental health problems, life-skills dimensions, and exposure to suicidal behavior in others were measured using the Youth Self-Report (YSR), Life-Skills Development Scale (LSDS)-Adolescent Form, and Attitude Towards Suicide (ATTS) questionnaires.

**Results:**

Suicidal plans were reported more often by teenage boys than teenage girls (M = 17.3%, F = 5.6%, p = 0.001), whereas girls reported more attempts (M = 0.6%, F = 7.8%, p = 0.012). Young men scored significantly higher on rule-breaking behavior than young women (p = 0.001), whereas young women scored higher on anxious/depression (p = 0.000), withdrawn/depression (p = 0.002), somatic complaints (p = 0.034), social problems (p = 0.006), and internalizing syndrome (p = 0.000). Young men exposed to suicide had significantly higher scores for internalizing syndrome compared to those unexposed (p = 0.001), while young women exposed to suicide scored significantly higher on both internalizing (p = 0.001) and externalizing syndromes (p = 0.012). Any type of exposure to suicidal expressions increased the risk for own suicidal expressions in both genders (OR = 2.04, 95% CI = 1.06-3.91); among young women, however, those exposed to suicide among friends and partners were at greater risk for the serious suicidal expressions (OR = 2.79, 95% CI = 1.00-7.74). Life skills dimension scores inversely correlated with externalizing syndrome in young men (p = 0.026) and internalizing syndrome in young women (p = 0.001).

**Conclusions:**

The significant gender differences in suicidal expressions and their determinants in Cambodian teenagers highlight the importance of culturally appropriate and gender-specific suicide prevention programs. School-based life skills promotion may indirectly influence the determinants for suicidal expressions, particularly among young women with internalizing syndrome in Cambodia.

## Background

Suicide is a global public health problem. An estimated 815,000 people worldwide took their lives in the year 2000, with an overall age-adjusted rate of 14.5 per 100,000 in the general population [1]. The suicide rate among young people has increased considerably over the last few decades in a number of high income countries, and the magnitude of the problem is 20 times higher when suicidal expressions are considered [2]. Suicidal expressions refer to life-weariness, death thoughts, suicidal ideation, suicide plans, and attempts, all of which are increasing among young people in many parts of the world. The National Co-morbidity Survey in the United States revealed suicidal ideation to be more common in the 18-24 age groups than in the general population [3]. Gender differences in suicidal expressions were insignificant in a study among high school students in US, with 13% of boys and 12% of girls reporting suicidal ideation [4]. In middle and low income countries, reliable figures on suicide rates are lacking and there are few studies on suicidal expressions. A hospital-based study from Nicaragua reported a higher incidence of suicide attempts among young women, with an estimated rate of 400/100,000 per year [5]. In Latvia, during the turmoil of the post-Soviet period, individuals between 18 and 30 years of age reported a high prevalence (53%) of any type of suicidal expression during the past year [6]. It is important to study the determinants of suicidal expression in order to prevent suicide among young people, particularly in post-conflict countries.

Among youth, about one-third of suicide victims satisfied the criteria for clinical depression or other treatable mental illnesses [7], underscoring the importance of research focused beyond the conventional risk factors such as exposure to suicide and psychosocial stressors [8]. A study among Lithuanian school students revealed that permissive attitudes towards suicide correlated with suicidal ideation and behavior [9]. A study that examined suicide ideation, behavior, and attempt history in 100 adolescents (age 17 to 19 years) found four factors to be important for overall suicide risk: hopelessness, hostility, negative self-concept, and social isolation [10]. These studies highlight the importance of understanding the mental health status of young people beyond clinical syndromes and other mental disorders. Suicide among young people and its impact on families and peers are major concerns for mental health professionals, school authorities, and service providers [11]. It is vital to understand the different forms of suicidal expressions among youth in relation to mental health status and exposure to suicide.

Exposure to suicide is a major risk factor for suicidal expressions [12]. A study among American Indian and Alaskan youth revealed that the most powerful risk factor for attempted suicide was having a friend who had attempted or committed suicide [13]. In addition to exposure to suicide and other well known risk factors, psychosocial problems and high-risk behaviors are associated with suicidal expressions among young people independent of psychiatric disorders [14]. Jessor et al. postulated a "syndrome of problem behavior" constituting delinquency, substance abuse, precocious sexual activity, and lack of social skills that were associated with suicidal expressions among young people [15]. Though some of the studies implicate life-skills and mental health competency as protective factors against suicidal expression among young people [16], there is no conclusive evidence on the type of intervention most efficacious in suicide prevention [17]. There is a wide 'research-gap' on the determinants of suicidal expressions among young people in low and middle income countries, particularly in post-conflict situations that are known to enhance the risk for mental health problems [18].

This study explores suicidal expressions and their determinants among high school students in Cambodia, a post-conflict country in economic, political, and social transition.

## Methods

### Setting

In Cambodia, there are 95 men per hundred women and 42% of the population is below the age of 15. The literacy rate is 85% for males and 64% for females. Eighty percent of the population of 14.5 million is rural and one sixth of the country's land is covered by landmines, a legacy of decades of war. The per capita gross national product (GNP) is 293 USD and 37% of the population lives in absolute poverty, earning less than a dollar per day [19]. Young people in Cambodia are vulnerable to high-risk behaviors and suicide due to the unstable economy, increasing unemployment, and lack of youth-specific services, consistent with research among transitional countries around the world [20]. Cambodian youth are at risk for traffic accident, HIV/AIDS, and drug abuse [21]. The trauma of the 'Pol Pot era' of the 1970s and subsequent two decades of civil strife have had repercussion on the psychosocial milieu, with significant impact on the young people. Substance abuse, sexual abuse, and early sexual experiences are common among young people in Cambodia [22] and all are known risk factors for suicidal expressions [23].

### Participants in the study

Three hundred and twenty students, aged 15-18 years in grades 10 and 11 from two high schools in Takhmau, a semi-urban area close to the Cambodian capital Phnom Penh took part in the study. All the students in four randomly selected classes in each high school were invited to participate and all 153 male and 167 females agreed.

### Instruments

#### Youth Self Report (YSR)

The YSR is a self-administered questionnaire that provides data on a broad spectrum of problems and competencies of young people in the 11 to 18 age group. It is a component of the Achenbach System of Empirically Based Assessment (ASEBA). The competency section is semi-structured, while the emotional behavioral section has 112 items with Likert-type scoring: 0-not true, 1-somewhat or sometimes true, 2-very true or often true. Based on empirical findings, the following syndrome scales are constructed, comprising problem-items that tend to occur together: *anxious/depression, withdrawn/depression, somatic complaints, social problems, thought problems, attention problems, and rule-breaking behavior*. The syndrome profiles are further coalesced into two broad syndromes, internalizing and externalizing [24]. The questionnaire is completed in 30 to 40 minutes and the responses refer to problems faced in the past six months. The Khmer version (Cambodian language) of the YSR was field-tested and updated based on a group discussion among psychologists and professionals working at the Center for Child and Adolescent Mental Health (CCAMH), Takhmau, Kandal province, Cambodia.

#### The "Attitudes Towards Suicide" (ATTS)

The ATTS is a semi-structured questionnaire with three sections. The first deals with exposure to suicidal expressions among significant others (parents, siblings, partners, relatives, and friends). The second section includes statements on attitudes towards suicide, and probes common beliefs and misconceptions about suicide. The third section queries the respondent's own suicidal expressions (life-weariness, death thoughts, suicide ideation, suicide plans, and suicide attempts) during the past year. The psychometric properties of the instrument were reported in previous studies [25,26]. The instrument was adapted to the local context after a series of discussions and translated into Khmer by mental health professionals at CCAMH. In this study, we focused on the first and third parts of the questionnaire; exposure to suicide attempts and completed suicide among significant others and their relation to own suicidal expressions.

#### Life Skills Development Scale (LSDS)-Adolescent Form

The LSDS-Adolescent Form is a 65 item instrument that measures four life skill dimensions: interpersonal communication/human relation skills, problem solving/decision making skills, physical fitness/health maintenance skills, and identity development/purpose in life skills. This self-administered questionnaire is also scored on a Likert-type scale by the responses completely agree, mostly agree, mostly disagree, or completely disagree. The reliability and validity of the LSDS-Adolescent Form has been established by previous studies [27,28]. The LSDS was translated and adapted to the Cambodian cultural context after focus group discussions with mental health professionals at CCAMH.

### Analysis

We performed bivariate and multivariate analyses using the SPSS statistical version 16. Chi-square tests were used to analyze frequency distributions and Student's t-tests were used to compare independent sample means. We dichotomized scores on the YSR and Life Skills Development Scale at the 90^th ^percentile for the multivariate logistic regressions model. Serious suicidal expression (plans plus attempts) was used as a dependant variable with gender, YSR syndrome, Life Skills Development domains, and exposure to suicide as covariates.

There were missing values on some YSR items among 11 participants (3.4%), and the values were replaced by medians of nearby scores.

### Ethical considerations

The directors of Chey Chumneas Hospital and the directors of the two high schools gave approval for the study. The teachers and the parents were informed about the nature of the study through the school administration and the parent association, respectively. We informed the students that participation was entirely voluntary and that they could opt-out at any time during the sessions. The issue of confidentiality was explained to the students before administering the questionnaires and they were not required to write their names. We informed the participants that confidential free-counseling services were available. Ethical clearance was obtained from the regional research ethics committee of Umea University, Sweden (Dnr: 07-046M).

## Results

### Suicidal expressions

Twenty-eight young men (17.9%) and twenty women (13.4%) reported serious suicidal expressions (plans plus attempts) during the past year, with no significant gender difference. Young men reported making suicidal plans more often than young women during the year prior to testing (M = 17.3%, F = 5.6%, p = 0.001), whereas attempts were more often reported by the young women (M = 0.6%, F = 7.8%, p = 0.012) (Table 1).

**Table 1:** Suicidal expressions during the past year among young people in Cambodia

ATTS Items	BOYS N = 157		GIRLS N = 163		TOTAL N = 320		Chi-square	
	N	%	n	%	N	%	**χ**^**2**^	p
Life not meaningful	138	87.9	140	86.4	278	87.1	0.156	0.693
Life-weariness	36	23.1	40	24.8	126	24.0	0.136	0.612
Death thoughts	39	25.0	41	25.6	80	25.3	0.016	0.898
Death wishes	22	14.1	27	17.0	49	15.6	0.497	0.481
Suicide ideation	15	9.6	20	12.3	35	11.0	0.605	0.437
Suicide plans	27	17.3	11	5.6	38	11.9	8.355	**0.001**
Suicide attempts	1	0.6	9	7.8	10	3.2		0.012*

*Fisher's exact test

### Mental health profile

Young men scored significantly higher on rule-breaking behaviour than young women, while young women scored higher on anxious/depression, withdrawn/depression, somatic complaints, social problems, and internalizing syndrome (Table 2).

**Table 2:** Mean scores on YSR syndromes by sex

	BOYS N = 157		GIRLS N = 163		t-test	
YSR Syndrome scales	Mean	SD	Mean	SD	t	P
Anxious/Depressed	8.45	3.59	10.11	4.14	3.184	**0.000**
Withdrawn/Depressed	4.16	2.49	5.06	2.69	3.133	**0.002**
Somatic Complaints	6.21	3.15	6.98	3.27	2.131	**0.034**
Social problems	6.29	3.34	7.26	2.90	2.769	**0.006**
Thought problems	5.86	3.44	5.35	3.53	1.293	0.197
Attention problems	7.71	3.05	8.33	2.66	1.196	0.056
Rule-breaking behavior	4.22	2.91	3.24	2.41	3.260	**0.001**
Aggressive behavior	8.24	3.76	9.08	4.29	1.875	0.062
Internalizing syndrome	18.82	7.41	22.14	8.50	3.726	**0.000**
Externalizing syndrome	12.45	5.93	12.32	5.93	0.195	0.845

Gender comparisons of YSR syndromes among those with and without serious suicidal expressions revealed that young men with serious suicidal expressions scored significantly higher on somatic complaints (p = 0.053) and internalizing syndrome (p = 0.021) than young men with no suicidal expressions. Young women with serious suicidal expressions scored higher on anxious/depression (p = 0.039), withdrawn/depression (p = 0.002), somatic complaints (p = 0.019), thought problems (p = 0.019), and internalizing syndrome (p = 0.004) than young women who did not report serious suicidal expressions.

Gender wise multivariate logistic regression with own serious suicidal expressions as the dependent variable and dichotomized YSR syndromes as covariates revealed that young women with anxious/depression (OR = 3.13; CI = 1.06-9.23) and internalizing syndrome (OR = 3.89; CI = 1.29-11.73) were significantly more likely to report serious suicidal expressions. There were no significant associations between any YSR syndromes and own serious suicidal expressions among young men.

### Exposure to suicide

Young women reported significantly more suicide attempts and completed suicides among friends or partners (p < 0.016) than young men.

Young men exposed to suicide attempts and completed suicide among significant others scored higher than young men without exposure on anxious/depression, withdrawn/depression, somatic complaints, social problems, thought problems, and internalizing syndrome. Young women exposed to attempts and completed suicide scored higher on all YSR dimensions, with the exception of social problems, compared to those not exposed (Table 3).

**Table 3:** Relation between YSR syndromes and exposure to suicide

	BOYS						GIRLS					
	Exposed to Suicide N = 31		Not exposed to suicide N = 126				Exposed to Suicide N = 41		Not exposed to suicide N = 122			
YSR Syndrome scales	Mean	SD	Mean	SD	t	p	Mean	SD	Mean	SD	t	p
Anxious/Depression	10.43	3.79	7.97	3.39	3.30	**0.002**	11.63	3.62	9.64	4.18	2.91	**0.005**
Withdrawn/Depression	5.67	3.13	3.78	2.17	3.18	**0.003**	6.22	2.91	4.67	2.51	3.06	**0.003**
Somatic Complaints	7.29	3.06	5.95	3.12	2.18	**0.034**	8.12	3.07	6.61	3.26	2.68	**0.009**
Social problems	7.45	3.59	6.01	3.22	2.05	**0.046**	8.05	3.23	7.00	2.75	1.87	0.066
Thought problems	8.29	3.84	7.45	3.59	4.08	**0.000**	6.64	3.58	4.93	3.43	2.67	**0.010**
Attention problems	8.51	2.37	7.52	3.19	1.93	0.058	9.29	2.48	8.03	2.64	2.75	**0.008**
Rule-breaking behavior	5.25	3.80	3.96	2.60	1.79	0.082	4.22	2.79	2.93	2.18	2.70	**0.009**
Aggressive behavior	9.10	3.86	8.02	3.73	1.40	0.169	10.26	4.29	8.69	4.25	2.04	**0.046**
Internalizing syndrome	23.39	7.95	17.70	6.85	3.66	**0.001**	25.97	7.69	20.93	8.39	3.55	**0.001**
Externalizing syndrome	14.35	6.83	11.99	5.62	1.78	0.082	14.48	6.32	11.62	5.65	2.56	**0.012**

Table 4 presents the analysis of exposure to suicide among different classes of significant others as related to own suicidal expressions. For both genders, being exposed to attempted or completed suicide within the immediate family (parents and siblings), among partners, or friends was significantly associated with own suicidal expression. When analysed by gender, young women exposed to suicidal behaviour among partners and friends were significantly more likely to have serious suicidal expressions, whereas there was no association between exposure to suicide and suicidal expression among young men.

**Table 4:** Relation between serious suicidal expressions (dependent variable) and exposure to suicide

	BOYS N = 157				GIRLS N = 163				BOYS and GIRLS N = 320			
Exposure to suicide or attempt	N	%	OR	95% CI	N	%	OR	95% CI	N	%	OR	95% CI
By parents or siblings	7	4.4	3.875	0.815-18.43	11	6.7	2.956	0.715-12.224	18	5.6	3.146	1.119-8.848
By partners or friends	16	10.2	2.417	0.765-7.643	30	18.4	2.786	1.003-7.738	46	14.4	2.380	1.127-5.027
By other relatives	24	15.3	1.304	0.440-3.864	15	9.2	1.103	0.230-5.290	39	12.2	1.302	0.538-3.150
By any group	37	23.6	2.200	0.904-5.353	44	27.0	1.963	0.743-5.184	81	25.3	2.039	1.062-3.915

### Life skills dimensions

Comparing scores on the four individual life skills dimensions as well as the total life skills score on the LSDS revealed that young men scored significantly higher on the human relations/interpersonal communication dimension (p = 0.001) and total life skills (p = 0.014) than young women.

There were several significant inverse correlations between life skills dimensions and mental health profile as revealed by the YSR (Table 5). Particularly, higher health maintenance/physical fitness skills inversely correlated with all YSR syndromes for both genders. The total life skills dimension score for both genders was inverse-correlated with all YSR syndromes other than thought problems, while there was a significant positive correlation with attention problems.

**Table 5:** Relation between LSDS dimensions and YSR syndromes (both sexes)-Pearson correlations

ITEMS	YSR syndrome scales										
LSDS Dimensions	Anxious/depression	Withdrawn/depression	Somatic complaints	Social problems	Thought problems	Attention problems	Rule-breaking behaviour	Aggressive behaviour	Internalizing problems	Externalizing problems	
Human relations/Interpersonal communication	-.084	.188**	-.100	.151**	-.061	-.104	-.004	-.017	-.141*	-.032	
Decision making/Problem-solving	-.076	-.121*	.029	-.114*	-.021	-.120*	-.011	-.116*	-.065	-.085	
Health Maintenance/Physical fitness	-.272**	-.301**	-.163*	-.289**	-.120*	-.261**	-.257**	-.306**	-.295**	-.327**	
Purpose in life/Identity development	.072	-.027	-.063	-.011	.126*	-.020	.017	.039	.001	.034	
All life skill dimensions	-.145**	-.258**	**-.124**	-.228**	-.033	.202**	-.119*	-.157**	-.203**	-.162**	

* p < 0.05, **p < 0.01,

When analysed by gender, the health maintenance/physical fitness dimension inverse-correlated with both internalizing syndrome (p = 0.021) and externalizing syndrome (p = 0.000) in young men. The total life skills score was inversely correlated with rule-breaking behaviour (p = 0.001) and externalizing syndrome (p = 0.026) among young men. Among young women, there were significant inverse correlations between health maintenance/physical fitness and both internalizing (p = 0.000) and externalizing syndrome (p = 0.000). Decision making/problem solving and total life skills scores were both inversely correlated with internalizing syndrome (p = 0.022, p = 0.001, respectively) and externalizing syndrome (p = 0.023, p = 0.049, respectively) in young women.

Internalizing syndrome among young women emerged as the only determinant having significant association with serious suicidal expression in the multivariate analysis (Table 6) using serious suicidal expression as the dependent variable and YSR-syndromes, life skills scores, and exposure to suicide as covariates.

**Table 6:** Multivariate analyses with serious suicidal expression as the dependent variable and exposure, YSR syndromes, and life skills dimension as covariates*

	BOYS N = 153				GIRLS N = 167			
	N	%	OR	95% CI	N	%	OR	95% CI
Exposure - family	7	4.4	3.389	0.561-20.478	11	6.7	2.965	0.569-15.440
Exposure - friend	16	10.2	1.165	0.298-4.549	30	18.4	2.032	0.644-6.413
Exposure - relatives	24	15.3	0.830	0.243-2.839	15	9.2	0.617	0.111-3.436
Internalizing**	-		1.071	0.996-1.152	-	-	1.103	1.023-1.190
Externalizing**	-	-	0.989	0.910-1.076	-	-	0.946	0.855-1.046
LSE total score**	-	-	5.622	0.604-52.340	-	-	0.234	0.021-2.612

*Adjusted model with all covariates entered.

**Internalizing, externalizing and total life skills entered as continuous variables.

## Discussion

We measured the prevalence of different suicidal expressions, including life weariness, suicidal ideation, plans and attempts, in a sample of young men and women in Cambodia, a low-income post-conflict country in social transition. We examined the associations between these suicidal expressions and mental health profiles, exposure to attempts/completed suicide in families or partners, and life skills, focusing particularly on gender differences. Significant gender differences emerged in the following aspects: the prevalence of the serious suicidal expressions (plan and/attempts), YSR syndrome scores, the reported frequency of exposure to suicide, life skills dimension scores, and the association between suicidal expressions and mental health profile. Among young women, internalizing syndrome significantly increased the risk for serious suicidal expression.

### Suicidal expressions

In our study group, 9.6% of males and 12.3% of females reported suicide ideation over the past year prior to testing, which is comparable to the frequencies reported by the study among high school students in US [5]. While the slightly higher reporting of suicidal ideation by young Cambodian women did not reach statistical significance, they did report significantly more attempts. In contrast, Cambodian young men reported more suicide plans. This contrasts to a community-based study in Nicaragua, where the females reported more death wishes [29].

The higher incidence of suicidal attempts among young women in Cambodia is comparable to findings from a study in India that reported three times more girls attempting suicide than boys [30].

*Mental health profile *also revealed significant gender differences, with young men scoring higher on rule-breaking behavior, while young women more often reported internalizing symptom, again in agreement with other studies [31,32] and [33]. There was also a gender difference in the association between suicidal expression and mental health profile. Among the young men, there was no significant association between serious suicidal expression and any of the YSR syndromes, while young women with anxious-depression and internalizing syndrome were more likely to report serious suicidal expression. This picture is slightly different from other studies, such as the one by Gould et al., where the boys with mood, disruptive, and substance abuse disorders more frequently reported suicidal expressions, while girls reporting suicidal expressions had significantly more anxiety and mood disorders [34].

### Exposure to suicidal behavior among significant others

To our knowledge, there are no previous studies exploring the association between exposure to suicide and suicidal expressions in low-income countries other than the Nicaragua study [29]. In Cambodia, significantly more young women reported exposure to suicide attempts or completed suicides among friends and partners, and there was a significant association between exposure and mental health problems in young women but not young men. This gender difference may reflect the readiness of young women to share information [35], while young men may consider it a weakness to disclose personal information [29]. Though both genders are likely to be influenced by exposure to suicide among any significant other, young women in Cambodia exposed to suicide among friends and partners were two times more likely to report serious suicidal expressions. This finding contrasts to other studies that failed to find any difference between exposed and unexposed adolescents [36].

### Life skills dimensions

In general, life skills are a less explored area in suicidology. In this study, young men scored higher on human relations/interpersonal communication and total life skills. For both the genders, heath maintenance/physical fitness and total life skills inversely correlated with most of the YSR syndromes. This highlights the importance of life-skills-competency in promoting mental health and preventing high-risk behavior among young people [37]. When analyzed by gender, a more complex picture emerged. Among the young men, total life skills inversely correlated with rule-breaking behavior and externalizing syndrome. The health maintenance/physical fitness dimension among young men inversely correlated with all YSR syndromes other than somatic complaints and thought problems.

Among the young women, heath maintenance/physical fitness inversely correlated with all YSR syndromes and, unlike young men, total life skills inversely correlated with both internalizing and externalizing syndromes. The complex associations and the dissimilarities among the genders in relation to life skill dimensions and YSR syndromes require further exploration in the context of gender-specific adolescent development [38].

Internalizing syndrome among young women remained significantly associated with serious suicidal expressions in the multivariate analysis with serious suicidal expression as the dependent variable and YSR-syndromes, life skills dimension scores and exposure to suicide as covariates, consistent with previous findings [39,40].

## Limitations of the study

Being a cross-sectional study, the associations do not reveal temporal relationships between suicidal expressions and the determinants under study. Furthermore, our findings from a semi-urban school may not necessarily generalize to the rest of Cambodia, which is predominantly rural. Some of the gender differences in suicidal expressions and their determinants among young people may emerge more robustly with a larger sample, stratified across rural, semi-urban, and urban settings.

## Conclusion

This school-based study revealed significant gender differences in suicidal expressions and their determinants among young people in Cambodia, highlighting the need for gender-specific suicide prevention strategies. Life skill dimensions and its relationship with adolescent suicidal expressions require further exploration by gender. A significant association between life skills problems and internalizing syndrome was found that in turn was associated with serious suicidal expression in young women. Promoting life skills in schools may enhance the overall mental health of young people in Cambodia [41,42], and indirectly influence the determinants of suicidal expressions, particularly among young women with internalizing problems.

## List of abbreviations

ASEBA: Achenbach System of Empirically Based Assessment; ATTS: Attitudes Towards Suicide; CCAMH: Center for Child and Adolescent Mental Health; LSDS: Life Skills Development Scale-Adolescent Form; YSR: Youth Self Report;

## Competing interests

The authors declare that they have no competing interests.

## Authors' contributions

BJ took part in the design of the study, carried out the data-collection and analysis, and drafted the manuscript. GK participated in the design of the study, performed the statistical analysis, contributed to the results section, interpretation of the data, and gave feedback on the manuscript. Both the authors have read and approved the final manuscript.

## Pre-publication history

The pre-publication history for this paper can be accessed here:

http://www.biomedcentral.com/1471-244X/11/47/prepub
